# Fungal infection and neurodevelopmental outcomes at 18–30 months in preterm infants

**DOI:** 10.3389/fped.2023.1145252

**Published:** 2023-04-20

**Authors:** Qi Zhou, Edmond Kelly, Thuy Mai Luu, Xiang Y. Ye, Joseph Ting, Prakesh S. Shah, Shoo K. Lee

**Affiliations:** ^1^ Department of Neonatology, Children’s Hospital of Fudan University, Shanghai, China; ^2^ Department of Pediatrics, Mount Sinai Hospital and University of Toronto, Toronto, ON, Canada; ^3^ Department of Pediatrics, Centre Hospitalier Universitaire Sainte-Justine and Université de Montréal, Montréal, QC, Canada; ^4^ Department of Biostatistics, Princess Margaret Hospital, Toronto, ON, Canada; ^5^ Division of Neonatal-Perinatal Care, Department of Pediatrics, Women and Children's Health Research Institute, University of Alberta, Edmonton, AB, Canada

**Keywords:** neurodevelopment, fungal, preterm, mortality, morbidity

## Abstract

**Background:**

Invasive fungal infection (IFI) is associated with significant mortality and morbidity among preterm infants but there has been no population-based study of long-term neurodevelopmental outcomes. The objective of this study was to examine population-based incidence trends as well as mortality, short term in-hospital morbidity and long-term neurodevelopmental outcomes among preterm infants with IFI, non-fungal infections (NFI) and no infections in Canada.

**Methods:**

We conducted a retrospective cohort study of 8,408 infants born at <29 weeks gestational age (GA), admitted to Canadian Neonatal Network neonatal intensive care units (NICU) from April 2009 to December 2017, and followed up at 18–30 months corrected age (CA) in Canadian Neonatal Follow-Up Network clinics. We compared mortality, long term neurodevelopmental outcomes and short term in-hospital morbidity among 3 groups of infants (IFI, NFI, and no infections).

**Results:**

The incidence of IFI was 1.3%, non-IFI 26.9% and no infections 71.7%. IFI incidence varied between 0.93% and 1.94% across the study period with no significant trend over time. Infants of higher gestational age were significantly (*p* < 0.01) less likely to have IFI. Among infants with IFI, NFI and no infections, the incidence of the significant neurodevelopmental impairment (sNDI) was 44.26%, 21.63% and 14.84% respectively, while mortality was 50%, 25.35% and 22.25% respectively. Even after risk adjustment for confounders (GA, Score for Neonatal Acute Physiology Version II, ruptured membranes >24 h, maternal antibiotic treatment, antenatal steroid use, cesarean section), infants with IFI had significantly higher odds of sNDI than NFI (aOR: 2.19; 95% CI: 1.23, 3.91) or no infections (aOR: 2.97; 95% CI: 1.55, 5.71), and higher odds of mortality than NFI (aOR: 1.55; 95% CI: 1.07, 2.26) or no infections (aOR: 1.45; 95% CI: 0.97, 2.17).

**Conclusions:**

Preterm infants with invasive fungal infections have significantly higher incidence of mortality and adverse neurodevelopmental outcomes than those with non-invasive fungal infections and no infections.

## Introduction

Disseminated or invasive fungal infections (IFI) is associated with significant morbidity and mortality among preterm infants; especially for very low birth weight (VLBW) newborn infants (1–4). It can involve virtually any organ or anatomic site, and can be rapidly fatal. Benjamin et al. (2) reported in a study from the National Institute of Child Health and Human Development (NICHD) Neonatal Research Network that as many as 9% of extremely low birth weight (ELBW) infants developed systemic candidiasis prior to hospital discharge. These infants were at significantly increased risk for death (32% compared with 17%; *p* < 0.05), moderate to severe cerebral palsy (CP, 14% compared with 6%; *p* < 0.05) and neurodevelopmental impairment (NDI) at 18 months of age, compared to infants without systemic candidiasis (3). Ballot et al. (5) also reported an increasing trend of neonatal fungal infections.

Ting et al. (6) recently reported that among infants admitted to neonatal intensive care units (NICU) in the Canadian Neonatal Network (CNN), the overall incidence of IFI was 0.22% among all admitted neonates, and 0.64% among infants <33 weeks gestational age (GA). Neonates with IFI had higher odds of mortality (adjusted odds ratio (aOR) 1.60 [95% confidence interval (CI) 1.06–2.43], necrotizing enterocolitis [NEC, aOR: 2.97 (95% CI: 1.76–5.01)], and severe retinopathy of prematurity [ROP, aOR: 2.15 (95% CI: 1.26–3.67)]. However, incidence trend and neurodevelopmental outcomes for extremely preterm infants with IFI in Canada have not previously been reported.

The objective of this study was to examine the trend of IFI incidence in Canada from 2009 to 2017, and to compare neurodevelopmental and growth outcomes of 3 groups of preterm infants born at <29 weeks' GA (with IFI (both early and late onset), infections without invasive fungal infections (NFI), and no infections) during hospitalization, at 18–30 months corrected age (CA). Although it is known that infants with infections have poorer neurodevelopmental outcomes than those without infections, we postulated that infants with IFI had the worst neurodevelopmental outcomes compared to infants with NFI and no infections.

## Design and methods

### Study design and population

We conducted a retrospective cohort study of all infants born at <29 weeks GA who were admitted to one of 26 tertiary-level neonatal intensive care units (NICU) participating in both the Canadian Neonatal Network (CNN) and Canadian Neonatal Follow-up Network (CNFUN) databases from April 1, 2009 to December 31, 2017 (7). We excluded infants who were moribund on admission, with major congenital anomalies, or missing birth date.

### Patient assessment and data collection

The CNN is a multidisciplinary collaborative research network involving all 32 tertiary-level NICUs in Canada. The CNN database includes information on maternal history, pregnancy and delivery, infant characteristics, outcomes and resource use, and has high reliability and internal consistency (8). Trained abstractors at participating NICUs prospectively abstracted data from patient charts into an electronic data system with built-in error checking and validated by separate audit, using a standardized manual of data definitions. The CNFUN is a national network of 26 neonatal follow-up clinics, and has a database built on and linked with the CNN database. It collects standardized data on neurodevelopmental outcomes for infants born at <29 weeks GA (9). Eligible patients were recruited and scheduled for an assessment at one of the CNFUN follow-up programs at a target age of 18–30 months CA, as outlined in the CNFUN manual (10). The assessment included a standardized history, physical exam, neurological exam for identification of cerebral palsy and the Bayley Scales of Infant and Toddler Development-Third Edition (Bayley-III) performed by trained personnel (11). For children with cerebral palsy, the Gross Motor Function Classification System (GMFCS) was used to determine motor functioning level (12, 13). Children who could not be tested were administered the Bayley-III Adaptive Behavior questionnaires. Hearing and visual function were ascertained based on audiology and ophthalmology reports. Data collection at each site was approved by either a research ethics board or quality improvement committees. Parental consent was obtained for the follow-up assessment. The current study was approved by the Executive Committee of the CNN and CNFUN and by the Mount Sinai Hospital Research Ethics Board in Toronto, Ontario.

### Outcomes and definitions

The primary outcome was significant neurodevelopmental impairment (sNDI), defined as having one or more of the following: CP with GMFCS ≥3, Bayley-III (cognitive, motor, language, adaptive) composite <70, need for hearing aids or cochlear implant, bilateral visual impairment; or severe global developmental delay as per clinician's impression in a child untestable on the Bayley-III (9).

Secondary outcomes included mortality (in NICU or after discharge but before 18–30 month follow-up), any NDI (defined as any of the following: CP; any Bayley-III composite <85, sensorineural or mixed hearing loss, or unilateral or bilateral visual impairment), each of the sNDI components, and growth (weight, length, head circumference <10th percentile for sex and age, according to the Centers for Disease Control and Prevention growth charts) (14). Other short-term NICU outcomes included seizure, severe neurological injury (SNI) (9), bronchopulmonary dysplasia (BPD) (15), ROP (>stage 2 or treated) (16, 17); NEC (≥modified Bell's stage 2) (18, 19); patent ductus arteriosus requiring ligation or surgical treatment (PDA).

IFI was defined as positive growth of fungus in the blood or CSF cultures, either early onset (culture positive in the first 48 h of life) or late onset (culture positive after 48 h of life). Fungemia only was defined as positive blood culture for fungi. Fungal meningitis only was defined as positive CSF culture for fungi but blood culture was negative. Fungemia with fungal meningitis was defined as positive blood and CSF cultures for fungi. NFI was defined as all infections except IFI, which include bacterium and/or virus infection, either culture proven or clinically suspected. No-infections was defined as no infection at all throughout the hospitalization. GA was defined as the best obstetric estimate based on early antenatal ultrasound, obstetric examination, and obstetric history. Severity of illness was measured using the Score for Neonatal Acute Physiology, version II (SNAP-II) as described by Richardson et al. (20).

### Statistical analysis

The study population was summarized descriptively. Trends of IFI rates were examined using the Cochran-Armitage trend test, as well as logistic regression analysis adjusted for possible confounders identified from univariate analyses and clinical experience. Infant characteristics were compared between IFI and NFI groups using Chi-square test or Fisher exact test for categorical variables, and Student t test or Wilcoxon rank sum test as appropriate for continuous variables. Differences in the primary and secondary outcomes between IFI and NFI groups were examined using the Chi-square test. Multivariable logistic regression analyses with adjustment for potential confounders were also conducted for long term neurodevelopmental outcomes, but not for short term neonatal outcomes because these outcomes may precede the onset of IFI. Data management and statistical analyses were performed using SAS 9.4 (SAS Institute, Cary, North Carolina). Two-sided *p* value of <0.05 was used to determine the statistical significance.

## Results

**Figure 1:**
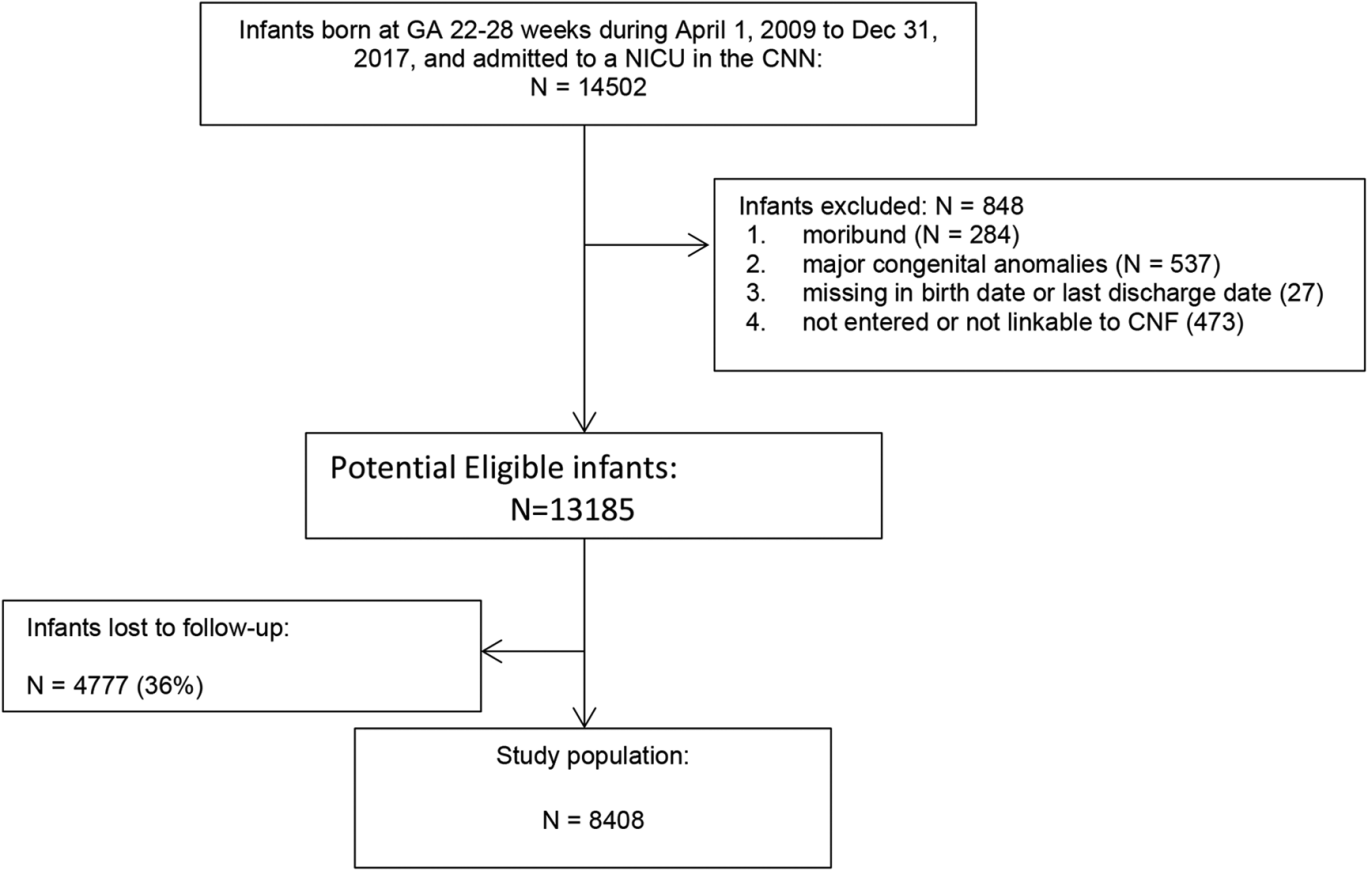
Flow chart diagram for study population.

Of the 14,502 infants born at <29 weeks GA, 848 were excluded (284 were moribund, 537 had major congenital anomalies, 27 had missing date of birth or discharge, 473 were not linkable between CNN and CNFUN databases) (Figure 1), 4,777 were lost to follow-up, and the remaining 8,408 infants comprised the study population. Compared to the study population, infants lost to follow-up had lower maternal age, and were more likely to have received maternal antibiotic treatment, be singleton, and have higher birth weight and GA, but less likely to be SGA, have Apgar scores <7 at 5 min, or SNAP-II scores >20 (Table 1).

**Table 1:** Comparison of characteristics between infants included in the study population and those lost to follow-up.

	Study Population	Lost to follow up	*p*-value
	(*N* = 8,408)	(*N* = 4,777)	
**Maternal characteristics**			
Maternal Age (years), mean (SD)	31.2 (5.82)	30.4 (5.96)	<0.0001
Diabetes, % (*n*/*N*)	9.0 (719/7,980)	8.7 (402/4,579)	0.66
Hypertension, % (*n*/*N*)	15.5 (1,271/8,192)	14.2 (663/4,640)	0.06
Rupture of membrane >24 h, % (*n*/*N*)	25.5 (2,120/8,299)	26.3 (1,250/4,745)	0.32
Maternal Antibiotic treatment, % (*n*/*N*)	67.6 (5,477/8,095)	71.9 (3,248/4,517)	<0.0001
Antenatal steroids, % (*n*/*N*)	87.7 (7,229/8,238)	88.5 (4,133/4,670)	0.21
Cesarean delivery, % (*n*/*N*)	57.3 (4,809/8,381)	56.7 (2,703/4,761)	0.5
Singleton, % (*n*/*N*)	72.6 (6,105/8,405)	75.1 (3,591/4,777)	0.002
**Neonatal characteristics**			
Birth weight (grams), mean (SD)	890 (245)	973 (256)	<0.0001
Gestational age (weeks), median (IQR)	26 (25, 27)	27 (25, 28)	<0.0001
Gestational age group, % (*n*/*N*)			<0.0001
≤25 weeks	38.4 (3,234/8,408)	26.8 (1,284/4,777)	
26–28 weeks	61.5 (5,174/8,408)	73.1 (3,493/4,777)	
Small for Gestational age, % (*n*/*N*)	9.5 (798/8,401)	6.5 (313/4,772)	<0.0001
Male, % (*n*/*N*)	54.5 (4,582/8,397)	54.4 (2,600/4,772)	0.93
Apgar Score <7 at 5 min, % (*n*/*N*)	44.1 (3,660/8,300)	39.6 (1,867/4,707)	<0.0001
SNAP-II score >20, % (*n*/*N*)	34.1 (2,817/8,244)	22.2 (1,058/4,755)	<0.0001
Infection type			<0.0001
No infection	71.6 (6,028/8,408)	77.2 (3,689/4,777)	
NFI	26.9 (2,264/8,408)	22.0 (1,053/4,777)	
IFI	1.3 (116/8,408)	0.7 (35/4,777)	

Notes: the reported *p*-values were based on the comparisons between two groups using *χ*^2^ test for categorical variables and student *t*-test or Wilcoxon Rank Sum test as appropriate for continuous variables.

SNAP-II, Score for neonatal acute physiology, version II; NFI, infections without invasive fungal infection; IFI, invasive fungal infection.

**Figure 2:**
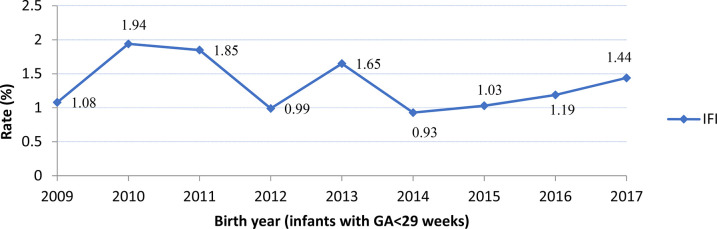
Rates of Invasive Fungal Infection (IFI) by year. No significant trend was observed (Armitage trend test: *p* = 0.27).

**Figure 3:**
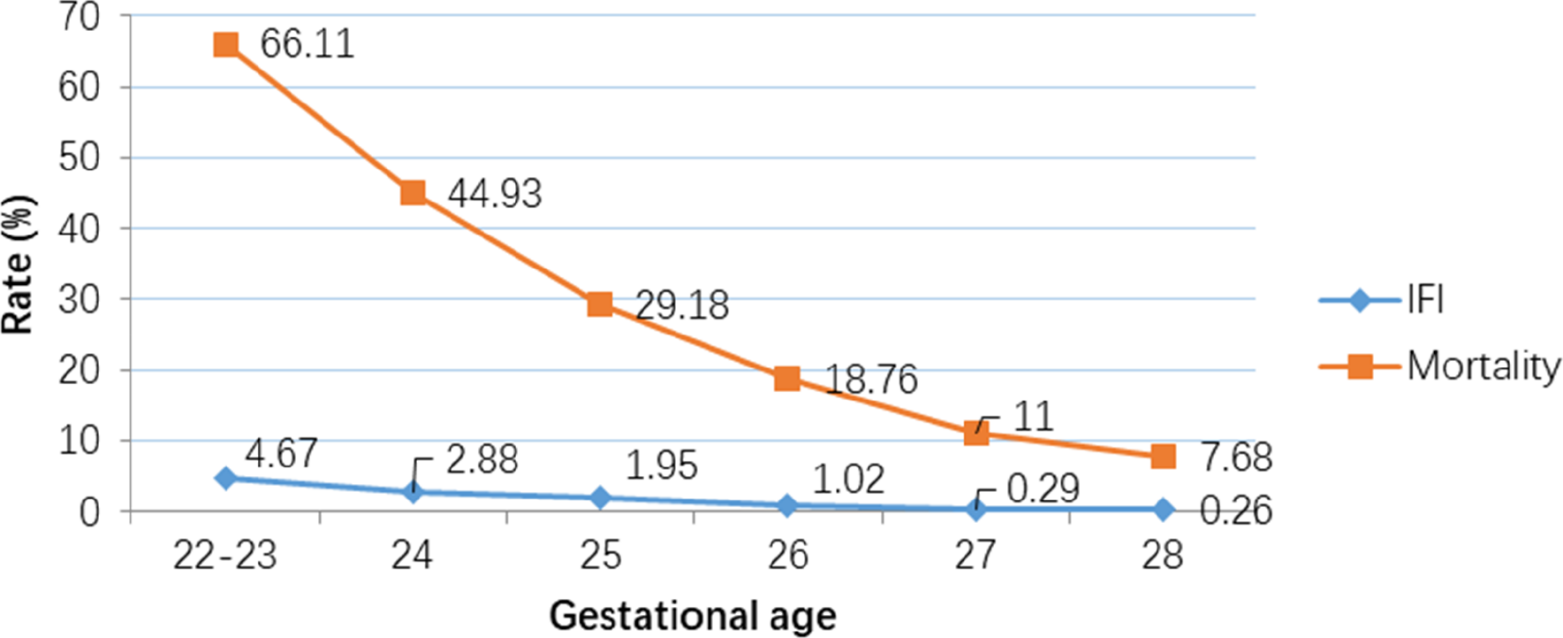
Decreasing rates of Invasive Fungal Infection (IFI) and mortality with increasing gestational age. (Armitage trend test: *p* < 0.0001).

The incidence of IFI was 1.3% (*n* = 116/8,408), NFI was 26.9% (*n* = 2,264/8,408) and no infections was 71.7% (*n* = 6,028/8,408) (Table 2). Compared to infants with no infection or NFI, infants with IFI had lower birth weight, GA, and lower rate of caesarean birth, but were more likely to have Apgar scores <7 at 5 min, SNAP-II scores >20, intubation at birth and receive surfactant treatment. There was no significant trend in IFI incidence (mean 1.34%, range 0.93–1.94%) between 2009 and 2017 (Figure 2). There was a significant trend (Armitage trend test *p* < 0.01) of decreasing IFI incidence with increasing GA, falling from 4.6% at 22–23 weeks GA to 0.2% at 28 weeks GA (Figure 3). The range of IFI across the sites were 0 to 6.9% (median = 2.0%) and 0 to 7.14% (median = 0) respectively for GA <26 weeks and GA 26–28 weeks' groups. Although there were only few IFI cases for each site [range:0–17 (median = 2)], there was variation in the rate of IFI across the sites (Fisher exact test: *p* < 0.0001), with range from 0 to 6.25% (median = 0.97%).

**Table 2:** Comparison of characteristics among no infection, infection without invasive fungal infection (NFI) and invasive fungal infection (IFI) groups.

	No infection	NFI	IFI	*p*-value
	(*N* = 6,028)	(*N* = 2,264)	(*N* = 116)	
	Including non-infection	Includes infection other than IFI (bacterium and/or virus infection)	Includes IFI only/IFI + bacteria or virus infection	
**Maternal characteristics**				
Maternal Age (years), mean (SD)	31.2 (5.81)	31.0 (5.82)	30.7 (6.16)	0.11
Diabetes, % (*n*/*N*)	9.3 (535/5,733)	8.2 (176/2,140)	7.4 (8/107)	0.27
Hypertension, % (*n*/*N*)	15.8 (929/5,858)	14.7 (328/2,223)	12.6 (14/111)	0.33
Rupture of membrane >24 h, % (*n*/*N*)	25.2 (1,502/5,954)	26.7 (597/2,232)	18.5 (21/113)	0.09
Maternal Antibiotic treatment, % (*n*/*N*)	66.9 (3,877/5,793)	69.5 (1,523/2,190)	68.7 (77/112)	0.08
Antenatal steroids, % (*n*/*N*)	87.1 (5,136/5,893)	89.5 (1,996/2,229)	83.6 (97/116)	0.005
Cesarean delivery, % (*n*/*N*)	58.4 (3,512/6,009)	55.4 (1,251/2,256)	39.6 (46/116)	<0.0001
Singleton, % (*n*/*N*)	72.4 (4,367/6,025)	73.2 (1,658/2,264)	68.9 (80/116)	0.53
**Neonatal characteristics**				
Birth weight (grams), mean (SD)	916 (250)	831 (218)	710 (158)	<0.0001
Gestational age (weeks), median (IQR)	26 (25, 28)	25 (24, 27)	24 (24, 25)	<0.0001
Gestational age group, % (*n*/*N*)				<0.0001
≤25 wks	33.1 (1,999/6,028)	50.5 (1,145/2,264)	77.5 (90/116)	
26–28 wks	66.8 (4,029/6,028)	49.4 (1,119/2,264)	22.4 (26/116)	
Small for Gestational age, % (*n*/*N*)	9.1 (553/6,023)	10.2 (232/2,262)	11.2 (13/116)	0.27
Male, % (*n*/*N*)	54.2 (3,268/6,021)	55.1 (1,247/2,260)	57.7 (67/116)	0.60
Apgar Score <7 at 5 min, % (*n*/*N*)	42.1 (2,508/5,946)	48.2 (1,080/2,239)	62.6 (72/115)	<0.0001
SNAP-II score >20, % (*n*/*N*)	31.7 (1,869/5,880)	39.1 (880/2,249)	59.1 (68/115)	<0.0001
Intubation at birth, % (*n*/*N*)	56.3 (3,040/5,399)	64.7 (1,274/1,967)	81.1 (86/106)	<0.0001
Surfactant, % (*n*/*N*)	73.3 (4,423/6,028)	82.1 (1860/2,264)	88.7 (103/116)	<0.0001

Notes: the reported *p*-values were based on the comparisons among three groups using *χ*^2^ test for categorical variables and student *t*-test or Wilcoxon Rank Sum test as appropriate for continuous variables.

SNAP-II, Score for neonatal acute physiology, version II.

Among infants with fungal infections, Candida species comprised 86% (*Candida albicans* 57.3%, *Candida parapsilosis* 20.5%, other Candida species 8.2%) and non-Candida fungal infections 14%.

There was a significant (*p* < 0.01) ordinal relationship of increasing incidence of mortality, and adverse long term neurological outcome (sNDI, mortality or sNDI, NDI, and Bayley III cognitive, language and motor scores <85), among the 3 infant groups with no infection, NFI and IFI. Among infants with no infection, NFI and IFI, the incidence of the primary outcome (sNDI) was 14.84%, 21.63% and 44.26% respectively, while mortality was 22.25%, 25.35% and 50% respectively. The weight, length and head circumference were correspondingly inverse (i.e., decreasing) among infants with no infection, NFI and IFI (Table 3). These differences persisted after adjustment for confounders using multivariable regression analyses (Table 4). After risk adjustment for confounders, infants with IFI had significantly higher odds of sNDI than NFI (aOR: 2.19; 95% CI: 1.23, 3.91) or no infections (aOR: 2.97; 95% CI: 1.55, 5.71).

**Table 3:** Comparison of long-term neurodevelopmental outcomes among no infection, infection without invasive fungal infection (NFI) and invasive fungal infection (IFI) groups.

	No infection	NFI	IFI	*p*-values
	(*N* = 6,028)	(*N* = 2,264)	(*N* = 116)	
Mortality or sNDI, % (*n*/*N*)	33.0 (1,993/6,028)	40.6 (921/2,264)	70.6 (82/116)	<0.0001*
Mortality, % (*n*/*N*)	22.2 (1,341/6,028)	25.3 (574/2,264)	50.0 (58/116)	<0.0001*
sNDI, % (*n*/*N*)	14.8 (704/4,743)	21.6 (371/1,715)	44.2 (27/61)	<0.0001*
NDI, % (*n*/*N*)	43.0 (2,042/4,743)	50.8 (872/1,715)	78.6 (48/61)	<0.0001*
Bayley's III cognitive score < 85, % (*n*/*N*)	13.5 (589/4,359)	19.4 (305/1,572)	30.9 (17/55)	<0.0001*
Bayley's III language score < 85, % (*n*/*N*)	34.5 (1,440/4,169)	40.0 (601/1,499)	62.9 (34/54)	<0.0001*
Bayley's III motor score < 85, % (*n*/*N*)	18.6 (776/4,159)	25.8 (387/1,499)	55.7 (29/52)	<0.0001*
Weight <10th percentile, % (*n*/*N*)	27.6 (1,237/4,481)	33.5 (553/1,647)	42.8 (24/56)	<0.0001*
Weight (*Z*-score), median (IQR)	−0.59 (−1.38, 0.18)	−0.82 (−1.59, 0.006)	−1.03 (−1.86, −0.08)	<0.0001*
Length <10th percentile, % (*n*/*N*)	53.8 (2,300/4,272)	59.7 (957/1,601)	68.5 (37/54)	<0.0001*
Length (*Z*-score), median (IQR)	−1.39 (−2.18, −0.63)	−1.57 (−2.46, −0.77)	−1.67 (−2.32, −0.98)	<0.0001*
Head circumference <10th percentile	17.5 (780/4,450)	27.8 (448/1,611)	35.8 (19/53)	<0.0001*
Head Circumference (*Z*-score), median (IQR)	−0.13 (−0.96, 0.69)	−0.59 (−1.37, 0.39)	−0.84 (−1.71, −0.02)	<0.0001*

Notes: the reported *p*-values were based on the comparisons among three groups using *χ*^2^ test for categorical variables and Wilcoxon Rank Sum test for continuous variables.

sNDI, significant neurodevelopmental impairment; NDI, neurodevelopmental impairment.

Mortality including deceased in the hospital and deceased after discharge.

* The rate of adverse outcome increased across the groups (no infection, non-IFI infection, IFI), assuming the classification was ordinal (Cochran-Armitage trend test: *p* < 0.0001).

**Table 4:** Multivariable regression analysis with adjustment for potential confounders, for comparison of mortality and long-term neurodevelopmental outcomes among no infection, infection without invasive fungal infection (NFI) and invasive fungal infection (IFI) groups.

	OR (95% CI)		
	NFI vs. No infection	IFI vs. NFI	IFI vs. No infection
Mortality or sNDI	1.15 (0.98, 1.35)	2.09 (1.36, 3.22)	2.41 (1.49, 3.89)
Mortality	0.93 (0.76, 1.14)	1.55 (1.07, 2.26)	1.45 (0.97, 2.17)
Significant neurodevelopmental impairment (sNDI)	1.36 (1.19, 1.56)	2.19 (1.23, 3.91)	2.97 (1.55, 5.71)
Neurodevelopmental impairment (NDI)	1.22 (1.11, 1.36)	2.86 (1.68, 4.89)	3.5 (2.13, 5.77)
Bayley's III cognitive score <85	1.31 (1.09, 1.58)	1.4 (0.79, 2.49)	1.84 (0.96, 3.53)
Bayley's III language score <85	1.15 (0.97, 1.38)	2.13 (1.35, 3.36)	2.46 (1.54, 3.94)
Bayley's III motor score <85	1.29 (1.13, 1.47)	2.7 (1.4, 5.21)	3.47 (1.75, 6.85)
Weight <10th percentile	1.20 (1.03, 1.39)	1.44 (0.87, 2.41)	1.73 (1.05, 2.84)
Length <10th percentile	1.14 (1.02, 1.27)	1.29 (0.76, 2.2)	1.47 (0.81, 2.65)
Head circumference <10th percentile	1.52 (1.32, 1.76)	1.31 (0.79, 2.19)	2.00 (1.22, 3.28)
		Difference in mean (95% CI)	
Weight (*Z*-score)	−0.12 (−0.18, −0.05)	−0.04 (−0.39, 0.32)	−0.15 (−0.52, 0.21)
Length (*Z*-score)	−0.12 (−0.19, −0.05)	0.07 (−0.30, 0.45)	−0.05 (−0.42, 0.33)
Head Circumference (*Z*-score)	−0.25 (−0.34, −0.16)	−0.18 (−0.44, 0.08)	−0.43 (−0.72, −0.14)

Notes: aOR (95% CI) = adjusted OR (95% CI) based on multiple logistic regression model adjusted for the potential confounders identified in the univariate analysis, including: gestational age, SNAPII score, rupture of membranes >24 h, maternal antibiotic treatment, antenatal steroid use and cesarean section.

sNDI, significant neurodevelopmental impairment; NDI, neurodevelopmental impairment.

Mortality including deceased in the hospital and deceased after discharge.

There was a significant (*p* < 0.0001) ordinal relationship of increasing incidence of adverse short term in-hospital outcomes (seizures, SNI, BPD, ROP, NEC and PDA requiring ligation or surgical treatment) among infants with no infection, NFI and IFI (Table 5).

**Table 5:** Comparison of short-neonatal outcomes among infants with no infection, infection without invasive fungal infection (NI) and invasive fungal infection (IFI) groups.

	No infection	NFI	IFI	*p*-values
	(*N* = 6,028)	(*N* = 2,264)	(*N* = 116)	
Seizure, % (*n*/*N*)	3.7 (202/5,464)	7.8 (157/1,994)	18.6 (20/107)	<0.0001*
SNI, % (*n*/*N*)	14.7 (826/5,592)	18.7 (416/2,224)	33.0 (38/115)	<0.0001*
BPD, % (*n*/*N*)	45.3 (2,147/4,737)	63.8 (1,135/1,778)	79.4 (54/68)	<0.0001*
ROP >stage2 or treated, % (*n*/*N*)	10.6 (401/3,773)	22.4 (368/1,642)	45.4 (30/66)	<0.0001*
NEC ≥stage 2, % (*n*/*N*)	5.9 (359/6,015)	16.4 (372/2,260)	24.1 (28/116)	<0.0001*
PDA required ligation or surgical treatment, % (*n*/*N*)	61.5 (2,076/3,371)	70.8 (1,161/1,639)	78.4 (73/93)	<0.0001*

Notes: the reported *p*-values were based on the comparisons among three groups using *χ*^2^ test.

IFI, invasive fungal infections; SNI, Severe neurological injury; BPD, Bronchopulmonary dysplasia; ROP, Retinopathy of prematurity; NEC, Necrotizing enterocolitis; PDA, Patent ductus arteriosus.

* The rate of adverse outcome increased across the groups (non-infection, non-IFI infection, IFI), assuming the classification was ordinal (Cochran-Armitage trend test: *p* < 0.0001).

## Discussion

To our knowledge, this is the largest and first population-based study of neurodevelopmental outcomes at 18–30 months CA, of preterm infants born at <29 weeks GA with fungal infections admitted to all tertiary level NICUs in Canada. Previous reports were from smaller non-population-based cohorts, and mostly compared IFI with non-fungal infections (combining no infection and non-IFI) or only with no infection controls (1, 2, 21, 22).

The IFI incidence of 1.3% in our study is similar to the 1.4% reported by Ting et al. (6) in an earlier cohort of EPI in Canadian NICUs, and to the 2.1% reported by Clerihew et al. (21) in a UK cohort, but lower than that reported by some large US cohorts (5%–9%) (2, 22, 23). Between 1997 and 2010, Aliaga et al. (23) reported a decreasing trend of IFI (from 8.2% to 2.3% for infants <750 g BW; from 2.4% to 1.1% for infants 750–999 g BW) associated with increased use of prophylactic fluconazole (from 0.3 to 11.0 per 1,000 patients for infants <750 g BW) in Pediatrix NICUs. In Canada, we have not noticed a change in trend of IFI incidence both in our study and the previous report by Ting et al. (6). Our observation of increasing IFI incidence with decreasing GA is also consistent with previous reports (2, 21–23), and reflects the diminished innate and reactive immunity, prolonged use of cannulas and catheters resulting in mucosal damage and integument disruption, and increased exposure to antibiotics altering the microbiome and resulting in overgrowth of fungi, among preterm infants (24).

Our finding that lower birth weight and GA, caesarean birth, Apgar scores <7 at 5 min, SNAP-II scores >20, intubation and surfactant treatment were risk factors for fungal infections is consistent with reports in the literature (2, 22). Other risk factors that have been described include use of broad spectrum antibiotics, cephalosporins, antacids (including histamine-2 blockers and proton pump inhibitors), mechanical ventilation and suctioning, delayed enteral feeding and parenteral nutrition (22).

Ngyuen et al. (25) reported the emergence of non-Candida albicans species and antifungal resistance in the 1990s, with non-*C. albicans* species accounting for half the Candida infections (23). This shift may be linked to the use of fluconazole prophylaxis as suppression of the predominant and virulent *C. albicans* subpopulation created space for less susceptible organisms. In our study, the incidence of *C. albicans* among infants with Candida infections was 57.3%, which is consistent with the 59.4% reported by Ting et al. (6) in an earlier Canadian cohort and merits monitoring.

Our results are consistent with previous reports showing higher incidence of adverse long term neurodevelopmental outcomes as well as adverse short term in-hospital outcomes among infants with IFI compared with NFI groups (1, 2). An ordinal relationship was observed with progressively lower incidence of mortality and adverse long term neurodevelopmental outcomes (sNDI, mortality or sNDI, NDI, and Bayley III cognitive, language and motor scores <85) among the 3 infant groups with IFI, NFI and no infection, and shows that IFI have significantly more profound impact on long term neurodevelopmental outcomes than NFI or no infection. These results are consistent with Benjamin et al.'s (2) report of higher mortality or sNDI with IFI from the NICHD Neonatal Research Network, and show that fungal infections can have a devastating impact on the long-term neurodevelopmental outcomes of preterm infants.

We also found a significant (*p* < 0.01) ordinal relationship of decreasing mortality and adverse short term in-hospital outcomes (seizures, SNI, BPD, ROP, NEC, PDA) among the 3 infant groups with IFI, NFI and no infection. This is only an association since some infections (NFI and IFI) may have occurred prior to the occurrence of the adverse short term in-hospital outcome. It is also possible that infants with adverse short term in-hospital outcomes are sicker and more prone to infection, with the sickest ones most susceptible to IFI.

There are several possible reasons why IFI may be more injurious than NFI. Fungal cells can invade tissues as they replicate, and disrupt cell function in multiple organs, including the central nervous system (22, 24). The immune response of the host is both innate and reactive (24). The innate system is the first line of defense, and utilizes multiple pathways to eliminate invading threats. The reactive system starts after the innate response and mainly utilizes cell mediated CD4+ and CD8+ T and B cells to coordinate a cytokine response. A dysregulated response can lead to a hyperinflammatory condition (cytokine storm), with neutrophils activation and may lead to organ dysfunction. An imbalance of this response can increase the anti-inflammatory response, leading to compensatory anti-inflammatory response syndrome (CARS), persistent inflammation-immunosupression, catabolism syndrome (PICS), and immune paralysis, which then leads to opportunistic infections by organisms like Candida. The host immune response is different for bacterial or Candida sepsis.

Prolonged use of even moderate doses of corticosteroids is common among preterm infants, and can be detrimental through both impairment of T-cell function and alteration of glucose metabolism (26). Fungi also exhibit competitive metabolism that consumes energy and nutrients intended for the host, and inhibit growth, repair and immunity (27). Fungi are more challenging than bacteria to treat without damaging the host because they are eukaryotes that share many of the same basic cell structures and machinery of animal cells. This can lead to off-target drug effects that may manifest as serious side effects in patients (28). Finally, while huge investments in antibiotic research have yielded many new drugs with improved efficacy and fewer side effects, this has not been the case with anti-fungal therapies. Indeed, Amphotericin B, which was first discovered in 1955, remains the mainstay of anti-fungal therapy today (29). Targeted antifungal prophylaxis in very high risk infants (e.g., 22–23 weeks GA requiring repeated and prolonged treatment with broad spectrum antibiotics, and prolonged parenteral nutrition *via* central catheter) might be of benefit, depending on the local IFI rate and microbiology data.

## Limitations

The follow-up rate in our study was only 63.7% of eligible infants, partly because of the Covid pandemic. However, infants who were not followed up had higher birth weight and GA, and less illness severity, and were therefore less likely to have fungal infections or adverse outcomes. Consequently, they are unlikely to lead to underestimates of the incidence of adverse outcomes. This study is an observational study which could not ascertain the cause-effect relationship inherently. Adverse short term in-hospital outcomes may occur before IFI and confuse the cause-effect relationship. Urine culture for candida was not routinely performed, which may have caused an underestimate of the IFI incidence. We did not have data on fluconazole prophylaxis use, which may underestimate rates.

## Conclusions

Preterm infants with fungal infections have significantly higher incidence of mortality and adverse neurodevelopmental outcomes than those with non-fungal infections and no infections, and there is an ordinal relationship of decreasing incidence among the 3 groups. Clinical practice improvements to prevent fungal infections in preterm infants and research to develop treatments with better efficacy and fewer side effects are urgently needed.

## Data Availability

The original contributions presented in the study are included in the article/Supplementary Material, further inquiries can be directed to the corresponding author.

## APPENDIX A

**Canadian Neonatal Network (CNN) Site Investigators**

Prakesh S Shah, MD, MSc (Director, Canadian Neonatal Network and Site Investigator), Mount Sinai Hospital, Toronto, Ontario; Marc Beltempo, MD, (Associate Director, Canadian Neonatal Network and Site Investigator), Montreal Children's Hospital at McGill University Health Centre, Montréal, Québec; Jaideep Kanungo, MD, Victoria General Hospital, Victoria, British Columbia; Jonathan Wong, MD, British Columbia Women's Hospital, Vancouver, British Columbia; Miroslav Stavel, MD, Royal Columbian Hospital, New Westminster, British Columbia; Rebecca Sherlock, MD, Surrey Memorial Hospital, Surrey, British Columbia; Ayman Abou Mehrem, MD, Foothills Medical Centre, Calgary, Alberta; Jennifer Toye, MD, and Joseph Ting, MD, Royal Alexandra Hospital and University of Alberta Hospital, Edmonton, Alberta; Carlos Fajardo, MD, Alberta Children's Hospital, Calgary, Alberta; Jaya Bodani, MD, Regina General Hospital, Regina, Saskatchewan; Lannae Strueby, MD, Jim Pattison Children's Hospital, Saskatoon, Saskatchewan; Mary Seshia, MBChB, and Deepak Louis, MD, Winnipeg Health Sciences Centre, Winnipeg, Manitoba; Ruben Alvaro, MD, and Ann Yi, MD, St. Boniface General Hospital, Winnipeg, Manitoba; Amit Mukerji, MD, Hamilton Health Sciences Centre, Hamilton, Ontario; Orlando Da Silva, MD, MSc, London Health Sciences Centre, London, Ontario; Sajit Augustine, MD, Windsor Regional Hospital, Windsor, Ontario; Kyong-Soon Lee, MD, MSc, Hospital for Sick Children, Toronto, Ontario; Eugene Ng, MD, Sunnybrook Health Sciences Centre, Toronto, Ontario; Brigitte Lemyre, MD, The Ottawa Hospital, Ottawa, Ontario; Thierry Daboval, MD, Children's Hospital of Eastern Ontario, Ottawa, Ontario; Faiza Khurshid, MD, Kingston General Hospital, Kingston, Ontario; Victoria Bizgu, MD, Jewish General Hospital, Montréal, Québec; Keith Barrington, MBChB, Anie Lapointe, MD, and Guillaume Ethier, NNP, Hôpital Sainte-Justine, Montréal, Québec; Christine Drolet, MD, Centre Hospitalier Universitaire de Québec, Sainte Foy, Québec; Martine Claveau, MSc, LLM, NNP, Montreal Children's Hospital at McGill University Health Centre, Montréal, Québec; Marie St-Hilaire, MD, Hôpital Maisonneuve-Rosemont, Montréal, Québec; Valerie Bertelle, MD, and Edith Masse, MD, Centre Hospitalier Universitaire de Sherbrooke, Sherbrooke, Québec; Caio Barbosa de Oliveira, MD, Moncton Hospital, Moncton, New Brunswick; Hala Makary, MD, Dr. Everett Chalmers Hospital, Fredericton, New Brunswick; Cecil Ojah, MBBS, and Alana Newman, MD, Saint John Regional Hospital, Saint John, New Brunswick; Jo-Anna Hudson, MD, Janeway Children's Health and Rehabilitation Centre, St. John's, Newfoundland; Jehier Afifi, MB BCh, MSc, IWK Health Centre, Halifax, Nova Scotia; Andrzej Kajetanowicz, MD, Cape Breton Regional Hospital, Sydney, Nova Scotia; Bruno Piedboeuf, MD (Chairman, Canadian Neonatal Network), Centre Hospitalier Universitaire de Québec, Sainte Foy, Québec.

**Canadian Neonatal Follow Up Network (CNFUN) Site Investigators and Steering Committee**

**Steering Committee:** Anne Synnes MDCM MHSc (Past Director), Thuy Mai Luu MD MSc (Director), Jehier Afifi MB BCh MSc (Co-Director), Rudaina Banihani MD, Jill Zwicker PhD OT, Lindsay Colby RN BScN MSN, Matthew Hicks MD PhD, M. Florencia Ricci MD PhD, Karen Thomas MD, Marie-Noelle Simard PhD, Lindsay Richter MSc (National Coordinator)

**Site Investigators:** Thevanisha Pillay MD, Victoria General Hospital, Victoria, British Columbia; Jessie VanDyk MD, British Columbia Children's Hospital, Vancouver, British Columbia; Rebecca Sherlock MD, Surrey Memorial Hospital, Surrey, British Columbia; Miroslav Stavel MD, Anitha Moodley MD, Royal Columbian Hospital, New Westminister, British Columbia; Leonora Hendson MD, Amina Benlamri MD, Alberta Children's Hospital/Foothills Medical Centre, Calgary, Alberta; Amber Reichert MD, Amy Shafey MD, Glenrose Rehabilitation Hospital, Edmonton, Alberta; Diane Moddemann MD MEd, Cecilia de Cabo MD, Winnipeg Health Sciences Centre, St. Boniface General Hospital, Winnipeg, Manitoba; Judy Seesahai MD, Windsor Regional Hospital, Windsor, Ontario; Sarah McKnight MD, Kingston General Hospital, Kingston, Ontario; Kevin Coughlin MD, Children's Hospital London Health Sciences Centre, London, Ontario; Linh Ly, MD, Hospital for Sick Children, Toronto, Ontario; Kamini Raghuram MD, Mount Sinai Hospital, Toronto, Ontario; Karen Thomas MD, Hamilton Health Sciences Centre, Hamilton, Ontario; Rudaina Banihani MD, Sunnybrook Health Sciences Centre, Toronto, Ontario; Kim-Anh Nguyen MD, Jewish General Hospital, Montréal, Québec; May Khairy, MD, Jarred Garfinkle MD, Montréal Children's Hospital, Montréal, Québec; Thuy Mai Luu MD MSc, Centre Hospitalier Universitaire Sainte-Justine, Montréal, Québec; Alyssa Morin MD, Centre Hospitalier Universitaire de Sherbrooke, Sherbrooke, Québec; Sylvie Bélanger MD, Centre Hospitalier Universitaire de Québec, Québec City, Québec; Jehier Afifi MB BCh MSc, IWK Health Centre, Halifax, Nova Scotia.
